# Developing a Theory-Informed Smartphone App for Early Psychosis: Learning Points From a Multidisciplinary Collaboration

**DOI:** 10.3389/fpsyt.2020.602861

**Published:** 2020-12-10

**Authors:** Natalie Berry, Matthew Machin, John Ainsworth, Katherine Berry, Dawn Edge, Gillian Haddock, Shon Lewis, Rohan Morris, Sandra Bucci

**Affiliations:** ^1^Division of Psychology and Mental Health, Faculty of Biology, Medicine and Health, School of Health Sciences, Manchester Academic Health Sciences, The University of Manchester, Manchester, United Kingdom; ^2^Division of Informatics Imaging and Data Sciences, Faculty of Biology, Medicine and Health, School of Health Sciences, University of Manchester, Manchester, United Kingdom; ^3^Greater Manchester Mental Health NHS Foundation Trust, Manchester, United Kingdom

**Keywords:** psychosis, smartphone, cognitive behavior therapy (CBT), co-design and co-production, public and patient involvement (PPI)

## Abstract

**Background:** Actissist is a smartphone app designed to deliver an intervention grounded in cognitive behavior therapy for early psychosis. Actissist was developed by a multidisciplinary team of academics, clinicians, experts by experience and software engineers. Actissist has been tested in two trials, the first a proof-of-concept trial where Actissist was safe, acceptable and feasible, the second, a powered randomized controlled trial.

**Objective:** This article describes how our multidisciplinary team designed and developed Actissist. This article describes: (i) how Actissist was informed by initial qualitative interviews and focus groups and an expert reference group; (ii) refinements made to the app based on ongoing user feedback; (iii) successes and challenges encountered; and (iv) learning points and recommendations for involving stakeholders in digital health interventions.

**Methods:** Expert reference group meetings informed the development of Actissist and design of subsequent trials, which included individuals with lived experience of psychosis, clinicians, academics, computer scientists and software engineers. Person-centered stakeholder involvement was promoted using focus groups and qualitative interviews prior to the development of the app, which informed version one of Actissist. Interviews were carried out with participants who had used Actissist. Two further versions of Actissist were developed following additional rounds of testing.

**Results:** Multidisciplinary working throughout the Actissist project led to the development, inclusion and improvement of the app design and content. These changes and features included non-directive and compassionate content, co-designed recovery videos, relaxation exercises, psychoeducation material, ability to “favorite” areas of the app that users found helpful, and goal-setting. Challenges to collaborative working included discrepancies between what stakeholders want and what is technically possible to deliver, resource pressures, trying to deliver desired features within the boundaries of fundamental trial design considerations, and power imbalances associated with multidisciplinary working.

**Conclusions:** The involvement of stakeholders in the design and development and delivery of Actissist has been fundamental to our development approach. Through this collaborative process, we have identified different perspectives and ideas that would have not been generated by the research team alone.

**Clinical Trial Registrations:** Proof-of-concept trial: http://www.isrctn.com/ISRCTN34966555

Fully-powered randomized controlled trial: https://www.isrctn.com/ISRCTN76986679

## Introduction

Psychological therapies are recommended to support individuals who experience psychosis, in addition to the provision of pharmacological interventions (1–3). Time-sensitive access to evidence-based interventions is important because a long duration of untreated psychosis is associated with a range of negative outcomes, including positive symptom severity, social functioning, quality of life and recovery (4, 5). Early intervention is recommended for people who have experienced a first episode of psychosis (6) and, in comparison with treatment as usual, is associated with better outcomes for treatment discontinuation, hospitalization, negative, positive and total symptom severity, and involvement at school and work (7). In accordance with clinical guidelines, early intervention services aim to provide time-sensitive access to a range of treatment options, including psychological therapy (8–10).

Despite evidence for the cost-effectiveness (11) and potential efficacy (12) of psychological support for early psychosis, access to such options are limited (13–15). Additionally, service users report a lack of choice in the decision-making process for treatment options, despite having strong feelings about how treatment should be delivered (16), resulting in feelings of disempowerment and disengagement (17). Therefore, novel technologies are now being used to scale up access to psychological interventions and offer greater choice for people who experience psychosis.

The provision of novel technologies to help support and deliver psychological interventions for people who experience mental health problems was a key topic in the recent Lancet Psychiatry commission by the World Psychiatric Association (18). Despite the potential of digital health interventions (DHIs), real-world usage rates are low (19). Lack of involvement of individuals with lived experience of mental health problems in the design of DHIs is a possible reason for these low usage rates (20).

The importance of stakeholder involvement in health research is increasingly being recognized with the creation of national standards and guidelines for public and patient involvement in research (21–24). Evidence of stakeholder involvement in the planning, administration and dissemination of projects is often cited on funders requirements for research grant applications. In the digital mental health domain, involving experts with lived experience is considered a top priority for DHI design by stakeholders including individuals with lived experience, healthcare professionals and academics (25–27). This collaborative approach can help identify different perspectives and ideas that would not necessarily be considered by the research team alone. To this end, stakeholder involvement has rightly been given a central role in the development of a wide range of DHIs including an electronic health intervention delivering psychoeducation and psychosocial support for careers of people with psychosis (28), a social media campaign for suicide prevention for young people (29), blending an app and face-to-face therapy for reducing paranoia in psychosis (30), a smoking cessation app for people experiencing serious mental health problems (31), mobile health solutions for individuals labeled as experiencing treatment-resistant schizophrenia (32) and social cognition training delivered via virtual reality for early psychosis (33). Although in its infancy, current evidence suggests that collaboration with service users may predict subsequent engagement with DHIs (34). Additionally, collaborative working between software engineers and clinicians is argued to be vital for ensuring that knowledge from multiple disciplines is used to inform DHI design (35) and avoid Type 1 (where designers do not accommodate user characteristics, preferences, context or needs) and Type 2 (where designers do not accommodate the clinical reality) design errors (36). In their person-centered approach to DHI design, Yardley et al. (37) also describe the need for the involvement of all relevant stakeholders, including service users and healthcare professionals.

Intervention development is a vital component of the UK Medical Research Council's (MRC) developing and evaluating complex interventions guidance. O'Cathain et al. (38) recommend that researchers detail the development process of their digital health intervention or technology to enhance understanding about the intervention development process to help: (i) readers understand the benefits and challenges of different intervention development approaches; (ii) readers select an intervention development approach that is relevant to their context; (iii) facilitate future retrospective assessment of how different intervention development approaches can lead to either effective or ineffective interventions that do or do not translate into practice change, and (iv) provide insights into research waste. However, this process is often not reported in an in-depth manner. In this paper, we describe a range of activities that we implemented to promote collaboration throughout the design and development of the Actissist app (39), a CBT-informed and self-guided smartphone app designed to help scale up access to CBT-informed information and strategies for people experiencing early psychosis. A proof-of-concept trial found that the app was safe, acceptable and potentially of clinical benefit for people with early psychosis (39) and a larger-scale RCT is currently in progress to explore its efficacy (40). When developing the Actissist app, detailed notes regarding multidisciplinary collaboration and the subsequent impact on app design and trial procedures were recorded, as recommended by Esmail et al. (41). Using the documented experiences of collaborative working on the Actissist project, this article describes the use of an expert reference group, beta-testing, qualitative interviews and focus groups with service users, clinicians, service managers and software and technology experts to inform the design of Actissist. We also provide an account of the challenges faced during the pursuit of multidisciplinary collaborative working and make recommendations for involving stakeholders in future digital health projects.

## Materials and Methods

Actissist (39) is a CBT-informed and self-guided smartphone app designed to help scale up access to CBT-informed information and strategies for people experiencing early psychosis. Actissist targets five domains associated with psychosis relapse: voice hearing, paranoia, perceived criticism, socialization and cannabis use. The five domains are accessed by selecting the “What's Bothering Me?” section of the app. Users work through a series of questions about the problem areas selected and are provided with hints and tips based on the information inputted. The app also contains a multi-media toolkit of activities and resources, including mindfulness and relaxation exercises, coping strategies, factsheets, a daily diary, recovery videos, goal-setting, graphical representations of symptoms and experiences and links to external content such as TED talks, blogs and mental health websites [see Bucci et al. (39) for more detail].

The Actissist app has been developed by a multidisciplinary team of clinicians, academics, software engineers and a User Interface (UI) designer. Each individual member of the team contributes expertise in areas that are fundamental to the development of the app and provides input into subsequent trials in which the app has been tested. The clinical research team is overseen by the principal investigator (a Professor of Clinical Psychology), with input from project co-investigators. Project co-investigators included three Professors of Clinical Psychology, a Professor of Adult Psychiatry, a Professor of Mental Health and Inclusivity, a Professor of Medical Statistics and Trials Methodology and a Professor of Health Informatics. The engineering team is overseen by the technical lead co-investigator (a Software Development Manager). The clinical research team are responsible for recruiting and working collaboratively with an expert reference group (ERG) of individuals with clinical, academic, technical and lived expertise in the fields of psychosis, and clinical research and/or app design and development. The clinical research team are also responsible for collecting and summarizing clinician and service user feedback regarding views toward the Actissist app. As well as actually implementing the app, the software engineering team provide guidance on the feasibility and effort required to implement the requested features. This influences both the prioritization of features and the way that they are implemented in order to provide a suitable balance between clinical need and what is technically feasible. Continued communication between the clinical research team and software team has been important to translate user ideas and feedback into creating and developing the Actissist app (42).

**Figure 1:**
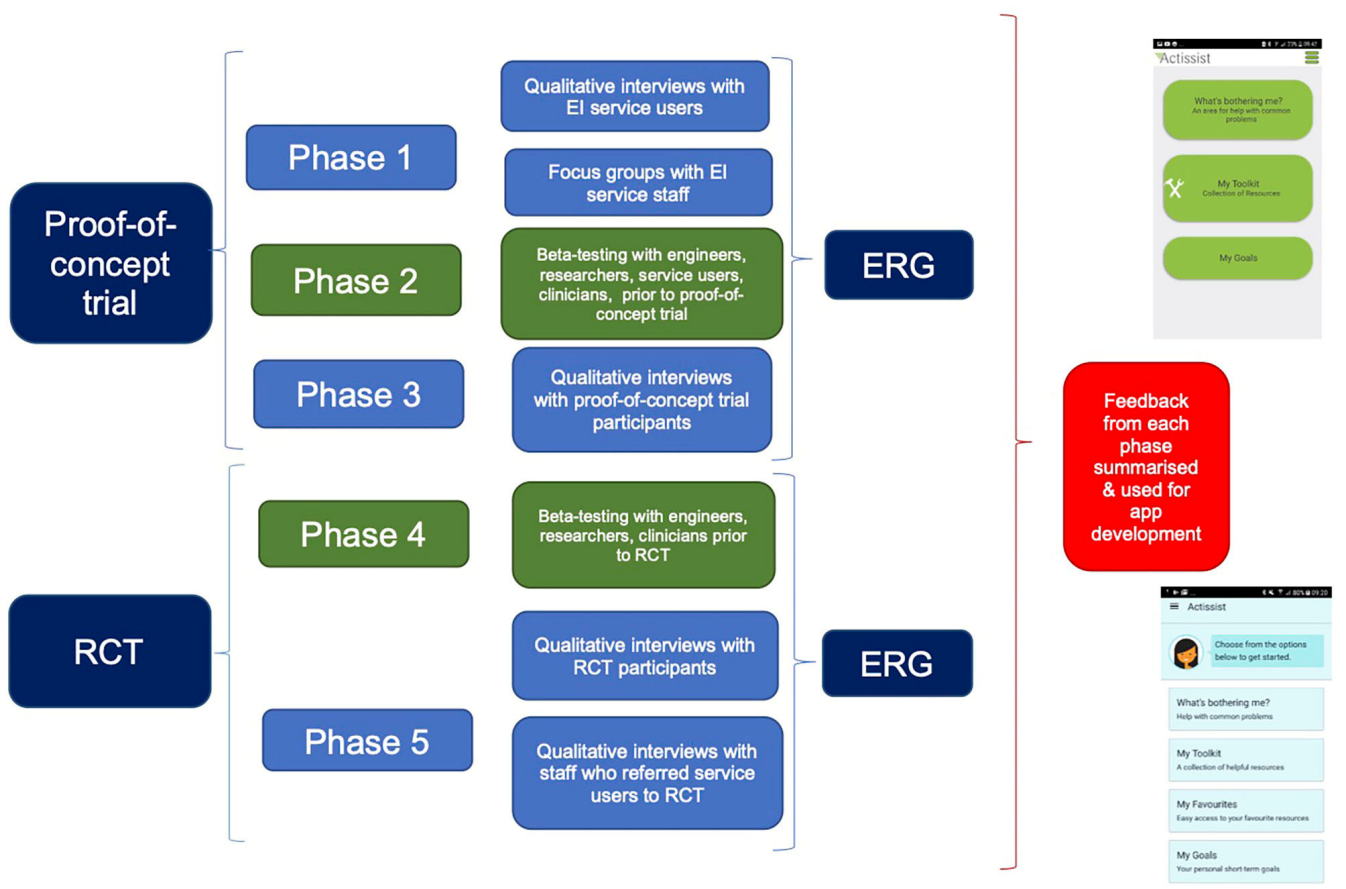
Process of stakeholder involvement during the design and development of the Actissist app.

The involvement of stakeholders in the development of the Actissist app and trial parameters is summarized in Figure 1. Over the course of 6 years, a range of stakeholder views have been sought, including individuals with lived experience (*n* = 54), clinicians (*n* = 65), software engineers (*n* = 3) and academics (*n* = 2). In phase 1, 21 qualitative interviews with service users and six focus groups with 48 clinicians were conducted to identify what they would want to see from a DHI designed for people with early psychosis. Participants were recruited through contacting service managers and clinical leads at NHS mental health trusts to send details about the qualitative interviews and focus groups to potential participants. Upon approval from team managers, members from the clinical research team attended NHS team meetings to inform clinicians about the clinician and service user studies and methods for referral.

Information gathered from phase 1, combined with a review of treatment manuals of CBT for psychosis (CBTp), was used to develop a prototype of the app which was beta-tested with five mental healthcare professionals and five service users for feedback on functioning, design and content (phase 2). Further refinements to the app were made by our software engineering team in response to feedback at which point version 1 of the app was developed and deployed in a proof-of-concept randomized controlled trial (39).

In phase 3, individuals who had participated in the Actissist proof-of-concept study were invited to take part in qualitative interviews about their experience of using the app. We have continued this iterative cycle of stakeholder views and feedback, app development, beta-testing (phase 4), trial testing and post-trial qualitative feedback throughout our DHI work. Since 2014, when the Actissist app development commenced, an ERG involving a range of stakeholders has convened over a quarterly basis to inform the design and content of the app and trial procedures.

### Qualitative Focus Groups and Interviews (Phase 1)

A series of six 90-min focus groups were conducted with clinicians working in early intervention services (EIS) in the North West of England in 2014. Focus groups comprised a total of 48 participants and included care coordinators, clinical psychologists, mental health practitioners, team managers, Support Time and Recovery (STR) workers, community psychiatric nurses, social workers, psychiatrists, researchers and a team secretary. In 2014, 21 semi-structured interviews were conducted with service users accessing EIS within the first 3 years of an initial episode of psychosis.

The topic guides (Appendix A in Supplementary Material) for both the clinician focus groups and service user interviews contained questions aiming to identify service user and clinician perspectives toward DHIs for early psychosis, including DHI design, features and content. Views and ideas expressed by service users and clinicians were identified during the data analysis phase and discussed with the software team and the clinical team responsible for the content development of the app. Full descriptions of the study methods and data analysis are published elsewhere (39, 42).

### Beta-Testing (Phase 2)

A prototype was developed based on service user and clinician ideas for app design and content elicited in phase 1. During phase 2, the app was tested internally within the engineering team (*n* = 5) and psychology researchers (*n* = 5) prior to beta-testing with five individuals who had experienced psychosis and seven clinicians working in secondary care services, who were asked to use the Actissist app for 1 week. Beta-test participants were recruited via emails to NHS mental health teams following permission from team managers. Feedback was elicited via semi-structured interviews using topic guides, which were audio-recorded and transcribed verbatim. Feedback from the beta-testing phase was sent to the engineering team to make the suggested changes to the app.

### Qualitative Interviews (Phase 3)

Participants who had received the Actissist app during the proof-of-concept trial were invited to participate in a qualitative interview at the end of the trial to describe their experiences of using the app. Fourteen participants subsequently consented to being interviewed. Although flexible and dynamic in nature, interviews were guided using a topic guide (Appendix B in Supplementary Material) and were audio-recorded and transcribed verbatim before being coded using framework analysis (43). Nvivo (version 11) was used to code the interviews. Data specifically relating to ideas about improvements and changes to the Actissist app were itemized using a template to highlight proposed changes to the app based on participant feedback. Participant feedback was discussed over several meetings with the engineering team using the information added to the suggestions for change template to aid discussion.

### Beta-Testing (Phase 4)

Upon receiving additional funding to trial the efficacy of the Actissist app, we further refined app content and functionality based on feedback received in phase 3. Decisions on which functionality to include in the app were taken collaboratively between the clinical research and software engineering teams based on a combined assessment of clinical benefit and technical feasibility. Members of our team (*n* = 4 engineers; *n* = 3 researchers) beta-tested the app over a seven-day period to ensure that the added and changed functionality worked as expected with no significant defects. The app was then further beta-tested with five clinicians working in mental health care settings who were independent to the project (*n* = 4 clinical psychologists; one clinical psychology researcher) to check app functionality. Problems with app functionality were sent to the engineering team who addressed the identified defects, carried out a full system test and then made the app available for use in the trial.

### Expert Reference Group (ERG)

Throughout our work, we have convened an ERG which has been fundamental to the design and development of the Actissist app and study procedures. ERGs were typically 90 min in duration and held on a quarterly basis over the course of our development work (2014–2020). All ERGs were conducted in a University building and facilitated by at least two members of the research team. Although most ERG members attended the meeting in person, video-facilities (e.g., Skype) were made available to enable participation for people who were not able to travel/attend in person.

ERG members: The number of people who participated in each ERG ranged from three to 11. Members were from a range of backgrounds including: individuals with lived experience of psychosis (*n* = 13); psychiatrists (*n* = 2); clinical psychologists (*n* = 2); software engineers (*n* = 3); a service user researcher (*n* = 1) and academics (*n* = 2). ERG attendees were recruited via patient and public involvement (PPI) groups in the Greater Manchester area, presenting ERG opportunities at local research events, a future contact consent form included in our trial procedures (see Appendix C in Supplementary Material) and emails to team managers working in NHS secondary care services. Attendees with lived experience were reimbursed for their attendance and travel expenses were also paid.

ERG process: Members were initially invited to attend ERG meetings via email, or by telephone if email contact information had not been provided. We asked members to complete an evaluation form at the end of the ERG meetings. A clinical psychologist was available in the event an ERG member became distressed as a result of the topics discussed during the meeting. Although this support option was available, no ERG members reported distress during or after any meetings throughout the 6 years of the project. Topics discussed at ERGs are presented in Table 1.

**Table 1:** Topics discussed at each expert reference group meeting.

**ERG attendees**	**Topics discussed**
Not recorded	Views about wording in the hints and tips section of the app. Discussion of items to include in the factsheets.
Not recorded	Detailed discussion regarding the themes proposed for the framework analysis of phase 1 clinician and service user qualitative interviews.
Not recorded	Views about the concept of therapeutic alliance with mental health apps. Ideas for the adaption of the therapeutic alliance measure used in the Actissist two trial.
Actissist team members (*n* = 3) Clinical academic (*n* = 1) Psychiatrist (*n* = 1) Service user (*n* = 3) Service user researcher (*n* = 1) Software engineer (*n* = 2)	Views about various sections of the app (e.g., wording of question-answer exchange items, mental health factsheets, coping strategies, relaxation and mindfulness exercises). Specific topics discussed include: • Terminology (is the language clear, should content be rephrased); • Content (does content work well and why, is there any content that does not work well and needs removing); • Usability (is the app easy to use, are there any technical problems); • App flow (do questions on the “What's Bothering Me” section of the app flow); • Aesthetics (is the app visually inviting to use; positive and negatives of the app design); • efficacy (does the app successfully incorporate CBT techniques, is the app likely to be helpful, is there anything about the app that might be unhelpful).
Actissist team members (*n* = 3) Clinical psychologist (*n* = 1) Data scientist (*n* = 1) Mental health nurse (*n* = 1) Psychiatrist (*n* = 1) Service user (*n* = 1) Software engineer (*n* = 1)	Views about overall app design/aesthetics. Views about app content and specific sections of the app. Feedback on trial recruitment procedures and ideas for participant recruitment. Feedback on the content of the participant information sheet.
Actissist team members (*n* = 2) Service user (*n* = 4)	Co-production of recovery journey videos for “Toolkit” section of the Actissist app. Members were asked to consider: • What did they experience during a FEP; • What support did they receive; • What coping strategies did they find helpful; • What they are doing now.
Actissist team members (*n* = 2)Data scientist (*n* = 1)Psychiatrist (*n* = 1)Service user (*n* = 2)Software engineer (*n* = 1)	Feedback on topic guide used for qualitative exit interviews (phase 5), specifically: • Phrasing (is it clear what each question is referring to); • Safety (are there any questions that may cause participants distress); • Relevance (are any questions not relevant/redundant); • Missing questions (are there any further questions we need to explore).
Actissist team members (*n* = 2) Data scientist (*n* = 1) Psychiatrist (*n* = 1) Mental health nurse (*n* = 1) Service user (*n* = 1) Software engineer (*n* = 1)	Feedback on recovery videos. Ideas for a new avatar for version 2 of the Actissist app due to negative feedback on the avatar from qualitative exit interviews (phase 3). Actissist website ideas.
Actissist team members (*n* = 2) Clinical psychologist (*n* = 1) Mental health nurse (*n* = 1) Software engineer (*n* = 1)	What ERG members would like to see included in an end of trial debrief form: • Links to further resources; • Summaries of other relevant papers; • Updates on trial progress; • Future research opportunities.
Actissist team members (*n* = 2) Data scientist (*n* = 1) Service user (*n* = 1) Software engineer (*n* = 1)	Feedback from previous participants about experiences of taking part in the trial to the group. Ideas for recruitment strategies to increase recruitment rates. Feedback on new avatar design for app version 2. Feedback on new design features for app version 2 (aesthetics; usability). Comments on the addition of: • An end of study debrief sheet for trial participants; • A future opportunities consent form to take part in future research and ERG meetings for participants to complete at the end of the trial; • Quarterly newsletters to presents updates about the trial. • Adaptions to the quantitative feedback questionnaire (QFQ).
Actissist team members (*n* = 3) Data scientist (*n* = 1) Service user (*n* = 2) Software engineer (*n* = 1)	Presentation of planned framework for the qualitative analysis of exit interviews. Feedback sought on: • Content of the themes; • Clarity of the themes; • Any additional themes that may be generated during analysis.
Actissist team members (*n* = 2) Software engineer (*n* = 1)	Review of the classification of technical-related adverse events and adverse reactions during the course of the Actissist RCT thus far.
Actissist team members (*n* = 2) Clinical psychologist (*n* = 1) Software engineer (*n* = 1)	Ideas to increase service user and mental healthcare professional ERG attendance. Feedback on recruitment methods for mental healthcare professional phase 5 qualitative interview nested study.
Actissist team members (*n* = 3) Clinical psychologist (*n* = 1) Data scientist (*n* = 1) Mental health nurse (*n* = 1) Psychiatrist (*n* = 1) Service user (*n* = 2) Software engineer (*n* = 1)	Co-analysis of quotations from qualitative interviews with participants who had received the Actissist app.

ERG feedback: Feedback from the ERG was presented to the Actissist investigator team and discussed as a group to understand improvements to the Actissist app design and content and trial procedures. A reporting template (see Appendix D in Supplementary Material) was completed after feedback was discussed to provide detailed recommendations for future refinements to the app and study procedures. Using a color-coded “traffic-light system” framework, the research team highlighted which suggestions were: (i) feasible to address in the short-term; (ii) potentially feasible to address in the short term; (iii) long-term/eventual changes; (iv) continually addressed as the project progresses; and (v) not possible to implement in the short- or long-term development of the app.

## Results

The results reported in this section focus on suggestions that were used to inform the design and content of the app and make revisions based on feedback. These findings have been compiled from the methods described above and personal reflections from the research team.

Full demographic information about participants who took part in the phase 1 and phase 3 qualitative work is available in Table 2. Service users who took part in phase 1 qualitative interviews were on average aged 26 years, roughly of equal gender, White British and largely owned a smartphone. Clinicians who took part in phase 1 focus groups were on average 39 years, were mainly female and White British, from a range of professional backgrounds but were predominately a care co-ordinator or clinical psychologist, and owned a smartphone. Service users who took part in phase 3 exit interviews were on average 27 years, mainly male and White British and owned a smartphone.

**Table 2:** Demographic characteristics for participants in qualitative interviews and focus groups.

	**Qualitative interviews—service users (Phase 1)**	**Focus groups–clinicians (Phase 1)**	**Exit Interviews–service users (Phase 3)**
Mean age (range)	26 (16–34)	39.2 (19–50)	27 (16–35)
Gender	Female: *n* = 11 Male: *n* = 10	Female: *n* = 27 Male: *n* = 20 Missing: *n* = 1	Female: *n* = 3 Male: *n* = 11
Ethnicity	Not recorded	White British: *n* = 40 Mixed: *n* = 4 White Irish: *n* = 1 Missing: *n* = 3	White British: *n* = 13 Black British: *n* = 1
Job title	Not applicable	Care coordinator: *n* = 10 Clinical psychologist: *n* = 8 CPN: *n* = 4 Mental health practitioner: *n* = 5 Psychiatrist: *n* = 4 Researcher: *n* = 2 Social worker: *n* = 4 STR worker: *n* = 5 Team manager: *n* = 5 Team secretary: *n* = 1	Not applicable
Mean time in post	Not applicable	3 years 8 months	Not applicable
Mean time working with EIS service users	Not applicable	5 years 4 months	Not applicable
Smartphone ownership	Yes: *n* = 18 No: *n* = 3	Yes: *n* = 42 No: *n* = 6	Yes: *n* = 12 No: *n* = 2

A summary of ideas for the Actissist app and feedback raised by participants, beta-testers and ERG members are presented in Tables 3–6 under the overarching headings: (i) technical features; (ii) app content; (iii) presentation of information; and (iv) procedural considerations. A number of technical features were identified as areas for development and improvement in the app (Table 3). These included type and number of alerts prompting participants to engage with the app, privacy and security features, and improvements in the design of the app and its usability. It was possible to integrate some of these features into the app, but resource limitations precluded some functionality improvements.

**Table 3:** Technical features identified as areas for development and improvement in the Actissist app.

**Technical feature**	**Phase identified**	**Description**	**Outcome**
**Alerts to engage with the app**			
Option of alerts	Phase 1 qualitative interviews Phase 1 focus groups	Alerts should be provided so people remember the app is there, but there should be the option to self-initiate use.	Alerts were included in the app, in addition to the option to self-initiate use.
Number of alerts	Phase 1 qualitative interviews Phase 2 beta-testing Phase 3 exit interviews	Mixed views about the number of alerts per day that should be sent (range: 1–8 per day). Tailoring the number and timing of alerts to user preference was suggested.	Tailoring the number and timing of the alerts was not possible due to the RCT design, but should be considered in future non-trial contexts.
**Privacy and security**			
Description of data confidentiality and security	Phase 1 qualitative interviews Phase 1 focus groups Phase 2 beta-testing	A disclaimer should be included in the app stating what happens to participant data.	A disclaimer was subsequently included in the “terms of use” section of the app and participant information sheet.
NHS and University affiliations	Phase 1 qualitative interviews Phase 2 beta-testing	People felt that they would trust the app more if there was a clear statement that the app was affiliated with the University and NHS.	Affiliations were included in the “about” section of the app.
**Operating system**			
Actissist should be made iOS compatible	Phase 2 and 4 beta-testing Phase 3 exit interviews	Actissist is compatible for all Android devices, but not iOS. Therefore, a common suggestion was to make Actissist iOS compatible.	Resource limits meant that it was not possible to make Actissist iOS compatible. However, iOS compatibility will be a priority for future iterations.
**Technical problems**			
Buttons	Phase 2 beta-testing	Beta-testers noted some of the buttons were unevenly spaced, not touch sensitive, difficult to see or did not direct users to the desired content. The sliding scale was difficult to navigate.	Errors with button functionality and appearance were resolved before running the proof-of-concept RCT. ERG members advised on adding numbers to the sliding scale to improve navigation for version 2 of Actissist.
Accessibility	Phase 4 beta-testing	One beta-tester commented that visual impairments made it difficult to see the text. A zoom function or option to enlarge the text was suggested.	Resource limits meant that it was not possible to improve accessibility before the main RCT. Improving accessibility will be a key consideration for future iterations.

**Table 4:** App content identified for including or revising in the Actissist app.

**Content**	**Phase identified**	**Description**	**Outcome**
**“What's bothering me?” section**			
Topics covered	Phase 1 qualitative interviews Phase 2 and 4 beta-testing Phase 3 exit interviews ERG	Majority were satisfied with the topics covered. Others suggested topic personalization or additional topics including: mood, anxiety, visual hallucinations, relationships, emotion regulation and substance use.	Resource limits in both the technical and clinical team meant that it was not possible to include additional topics in this section due to the complexity of the branching structure to receive hints and tips. Therefore, the additional topics suggested were instead included as factsheets in the “My Toolkit” section.
Identifying what is bothering them	Phase 3 exit interviews ERG	Participants and ERG members said it might be difficult to identify how they were feeling at the time of being alerted and suggested the inclusion of a “none of the above,” “don't know” or “other” response option.	An “other” option was subsequently included in version 2 following further feedback from the ERG.
**Daily diary**			
Editing and deleting diary entries	Phase 3 exit interviews	Two participants were frustrated they were unable to delete or edit diary entries.	We had to select changes based on what was prioritized by participants and ERG members due to resource and time limits. Some participants in the exit interviews stated that they did not use the daily diary; therefore, other suggestions for changes were prioritized.
**Psychoeducation factsheets**			
Topics covered	Phase 1 qualitative interviews Phase 2 beta-testing	Topics suggested were: (i) symptoms and experiences associated with psychosis; (ii) self-esteem; (iii) normalizing information (for example, statistics about psychosis prevalence); (iv) emotion-regulation; (v) anxiety; and (vi) evaluating thoughts.	Suggested topics were included in the factsheets in version 1.
Information overload	Phase 3 exit interviews ERG	Whilst the information included was considered helpful, some felt it was too much information and suggested an expansion button could be included to view additional information.	A UI designer was commissioned to improve the visual appearance and display of information in the factsheets for version 2 of the Actissist app.
**Relaxation and mindfulness exercises**			
Inclusion of relaxation and mindfulness exercises	Phase 1 qualitative interviews	Many people suggested the inclusion of more exercises; however, 3 service user participants stated they would be concerned that the app would “get bogged down with too many features.”	Actissist is CBT-informed so improvements in CBT content were prioritized. Further external links to mindfulness-based content were included in the additional resources section of the app based on this feedback.
**Recovery story videos**			
Character diversity	Phase 2 and 4 beta-testing ERG	Characters were limited to males. Additional recovery videos with more diverse demographic characteristics should be created.	Additional funding was secured to create two further recovery videos, which promoted more diversity in characters. These videos were included in version 2 of the app.
**Graphical summaries**			
Graphical summary explanation	ERG	ERG members suggested including an explanation on the graphical summaries that normalizes symptom fluctuations and advises users should speak to someone they trust if they are worried about the information presented on the app.	A detailed explanation of the graphs, normalizing information and signposting for advice were subsequently included in version 1 of the app.
**Goal-setting**			
Inclusion of a goal-setting function	Phase 3 exit interviews ERG	Participants and ERG members suggested including a goal-setting function, which could include reminders to help users accomplish goals.	Prioritized and included in version 2 of the Actissist app due to goal-setting being an important component of CBT.

**Table 5:** Ideas and changes relating to the presentation of information in the Actissist app.

**Presentation of information**	**Phase identified**	**Description**	**Outcome**
**Avatar**			
Visual appearance	Phase 3 exit interviews	Some participants felt the avatar was not visually appealing and could be improved.	UI designer commissioned to create a more visually appealing avatar for version 2.
**Favoriting items**			
Option to favorite items	Phase 2 beta-testing Phase 3 exit interviews ERG	Concerns were raised that the volume of information would leave users unable to find information or exercises they had found helpful.	An option to favorite items was included in version 2 of the app.
**App appearance**			
Basic design	Phase 3 exit interviews	Participants felt the Actissist app design and appearance was quite basic. Participants wanted to be able to personalize the appearance more and requested that the app was more colorful and inviting.	Personalization options were added in version 1. A UI designer was commissioned to improve the look-and-feel of the app for version 2.
Text-based	Phase 3 exit interviews	Additional multimedia options for viewing app content were suggested such as videos and audio files.	Space on the app, resources available and the technical complexity involved in including additional multimedia options meant that it was only possible to deliver two additional recovery videos.
**Content phrasing**			
Button titles/response options not accurately reflecting content	Phase 2 beta-testing Phase 3 exit interviews ERG	The following buttons and responses were viewed as not accurately reflecting the content included: “How am I doing”; “How am I feeling”; “Disclaimer”; “Helpful numbers”; “How do you feel”; “I can't be bothered”.	The following changes were made to version 2: “How am I doing” changed to “my progress.” “How am I feeling” changed to “how have I been feeling.” “Disclaimer” changed to “terms of use.” “Helpful numbers” changed to “helpful contacts.” “How do you feel” changed to “choose the option that is closest to how you are feeling.” “I can't be bothered” changed to “I feel like I can't be bothered.”

**Table 6:** Procedural considerations identified for the overall Actissist project.

**Procedural considerations**	**Phase identified**	**Description**	**Action/outcome**
**Information/training for app use**			
Video- and paper-based instructions	Phase 2 beta-testing ERG	Paper instructions and a video tutorial highlighting how to use the app was suggested to help promote engagement.	Paper instructions were given to participants during a phone set-up session where participants were shown how to use the app. Resource limits meant we did not have time to film a video tutorial.
**Trial measures**			
Stigma measure	Phase 1 qualitative interviews Phase 1 focus groups ERG	Participants and ERG members suggested apps could be destigmatizing and normalizing.	A measure of internalized stigma was included in the main RCT.
**Identifying participant feedback**			
Qualitative interviews & ongoing feedback	Phase 3 exit interviews	Some participants struggled to remember specifics about the app after their 22-week follow-up assessment. One participant suggested the option to text, email or write in the app ongoing feedback to help gather more information about peoples' views.	Suggestion for ongoing feedback not included in the main RCT due to the potential to be detrimental to participants focusing on other areas of the app, thus potentially impacting study outcomes.
Topic guide development	ERG	ERG members advised on the development of the topic guide. A key recommendation was exploring the key components of the app which yield a therapeutic benefit to the user.	ERG members approved the topic guides prior to interviews and important additions were made based on their feedback.
**Participant recruitment**			
Strategies to aid recruitment	ERG	ERG members advised on multiple strategies to reach participants including: approaching teams to contact service users on CBT waiting lists and offering training incentives.	Suggestions by the ERG were implemented and the trial recruited to target within the specified time limit.
**Involvement of healthcare professionals**			
Automatic transfer of data	Phase 1 qualitative interviews Phase 1 focus groups Phase 3 exit interviews	Actissist is a self-guided intervention and does not require input from a healthcare professional; however, some participants felt it would be valuable for clinicians to have access to this data to help inform practice and support.	Feedback regarding automatic transfer of data was mixed and clinicians may respond to app data in different ways, thus affecting study outcomes. Therefore, we decided against sharing app data with clinicians.
**Expert reference group**			
ERG evaluation form	ERG	After the proof-of-concept study, the research team reflected that more information could have been gathered about ERG member experiences.	An evaluation form was created and completed by ERG members after each meeting during the main RCT period. Comments were used to organize and inform subsequent ERGs; however, no formal analysis took place.
ERG dynamics	ERG	Whilst steps were taken to reduce potential power imbalances, research assistants reflected sometimes feeling intimidated by the seniority of some attendees, although this view was not expressed by ERG members.	Future formal evaluations of ERG meetings are encouraged to explore views toward power dynamics.
ERG member attendance	ERG	Three ERG meetings did not have service users in attendance.	Topics were presented again at subsequent ERGs and feedback was sought on how to improve attendance, including changing the time and offering virtual methods of attendance.

Participatory involvement over the four phases helped identify and refine app content, wording and terminology (Table 4). Ongoing engagement with an ERG throughout the project resulted in additional features included; for example, the inclusion of an “Other” domain in the event domains available for selection were not relevant for the participant. Links to mindfulness content and additional recovery story videos, were also added. Qualitative interviews with participants who had used the app ensured that improvements including the provision of a goal-setting function, changes to terminology, and the inclusion of further factual information were made to improve future engagement with the app (Table 4). Phase 3 exit interviews primarily influenced ideas and changes relating to the presentation of information in the app (Table 5). A UI designer was recruited to improve features such as the avatar in the app, an option to favorite items, and improvements were made to the overall look-and-feel of the app. The ERG also influenced trial procedures and materials. For example, an additional measure to capture stigma was included in study assessment measures, alterations to qualitative interview topic guides, strategies to aid recruitment and to improve the process of running the ERG were incorporated as the project evolved (Table 6).

## Discussion

The development of the Actissist app and subsequent trials have been shaped by the involvement of stakeholders throughout the design and delivery process. A person-centered design approach (37) of using qualitative interviews and focus groups with stakeholders prior to designing the app facilitated the inclusion of the recovery videos and additional factsheets that had not been incorporated in initial prototypes of the app. Where possible and feasible, changes were made based on stakeholder feedback throughout the lifecycle of the project. However, whilst elements of the involvement process were successful, challenges were also present throughout.

### Successes and Challenges

#### PPI Involvement Group vs. ERG

A PPI group involves individuals with lived experience of the condition under consideration or members of the public, whereas an ERG is attended by all relevant stakeholders. The ERG format was chosen due to unique insights that a differing range of individuals could provide with regards to technical input, content and trial design. We were mindful of the potential power imbalance that could be created through having a range of individuals with different experiences in attendance. In pursuit of collaboration rather than consultation, we hoped to facilitate a meeting which allowed researchers, clinicians and service users to have equal contributions to the research process. However, it was often challenging to maintain service user involvement throughout the course of the project. As such, there were three ERG meetings not attended by service users where feedback was solely gathered from clinicians, software engineers and researchers. In these cases, the topics covered were presented again at subsequent ERG meetings. We also sought advice from ERG members about methods to improve attendance at future meetings. Suggestions such as changing the time from the morning to the afternoon, collaborating with service users to arrange dates rather than offering pre-specified times and offering the opportunity to attend via teleconferencing were taken on board and subsequently improved attendance.

A strength of the ERG was that it allowed us to facilitate a group where members had equal importance in the design and development process of both the app and the trials. However, some individuals who have accessed mental health services experience compulsion and coercion within services and over-medicalization of distress. It has been argued that such dynamics within services can manifest when involving individuals in research, which leaves those in power positions questioning the perceived legitimacy of contributions (44). Furthermore, individuals themselves may also feel that they do not have a legitimate voice or valued contribution when in a room of professionals. To try to mitigate such power imbalances and ensure meaningful collaborations between researchers, software engineers, service users and clinicians, we tried to ensure that the language used was acceptable to all and any acronyms fully explained. Ground rules presented at the start of ERG meetings facilitated awareness that each member had valuable views to offer and should be given the opportunity to speak. Although the chair at the meeting stood up to present the slides, the co-researcher sat amongst members to create a collaborative dynamic and watch group interactions to allow them to intervene if they noticed an ERG member wanted to raise a point. Although previous research has highlighted the need for a researcher to act in the “leader” role, it has been suggested that individuals with lived experience could take on the role of co-delivery (45). We considered asking a member with lived experience to co-chair ERG meetings to adjust the potential power imbalance that can occur due to the multidisciplinary nature of the ERG; however, the lack of consistency in attendees meant that it was unfortunately not possible to identify someone to take on that role.

For the majority of meetings, the ERG comprised individuals from multidisciplinary backgrounds; however, a service user-only meeting was facilitated for the co-creation of recovery story videos. The decision to take a service user-only approach was to ensure that the videos created were directly relevant to individuals' recovery experiences, rather than based on professionals' perceptions. Retrospectively, the same approach could have been taken for the discussion of other aspects of the project. For example, specific technical issues could have been discussed within a software engineering-only group, analysis of service user qualitative interviews could have been undertaken with a service user-only group, whilst strategies to discuss recruitment within NHS service settings could have involved a clinician-only group. Therefore, whilst it was important to promote multidisciplinary attendance at many of the ERG meetings, researchers should also consider early in the planning stages additional smaller subset groups at certain points of the project.

#### Evaluation

Members of the ERG were provided with a short evaluation form to complete after each meeting. The option to receive a telephone call after the ERG was also included later in the main RCT so members were able to reflect on their experiences. Feedback from each ERG meeting was discussed within the Actissist team and improvements made based on suggestions. On reflection, we also would have sought ethical approval and consent from ERG members for the analysis and publication of individual experiences of taking part in the ERG. This would have allowed us to identify reasons for non-attendance and determine whether membership was helpful, or indeed unhelpful, for individuals in terms of their own outcome, development, interest and well-being.

#### Trial Procedures

The ERG contributed toward several important procedural features and considerations for the proof-of-concept RCT and main RCT. For example, ERG members suggested that a key focus of the qualitative exit interviews should be *how* the Actissist app has been helpful or unhelpful for participants to explore potential mechanisms of change. The completed topic guide was subsequently presented at later ERG meetings for feedback on contents and phrasing. As such, a strength of our qualitative work has been the involvement of stakeholders in its development. However, one drawback of this process is that we were unable to truly co-produce an analytical process with relevant stakeholders due to time and resource constraints. Researchers are increasingly involving stakeholders in the delivery of qualitative interviews, the coding of transcripts, co-creation of themes and dissemination of the results (46). Involving relevant stakeholders in interview delivery can result in deeper and more personal insights due to the rapport and empathy that can be generated by a shared understanding (47). Without meaningful collaboration in qualitative studies there is also a risk of bias from viewing data from a purely clinical academic perspective (48). Although the provision of feedback from the ERG was a key strength of the overall project, in future we would want to ensure funding was available to collaborate with relevant stakeholders in the delivery and analysis of qualitative interviews for such projects.

A key consideration for the RCT process was the measures used to identify outcomes. Often these decisions are based on factors such as psychometric properties and previous literature to ensure validity and study comparability (49). The measures chosen for the Actissist proof-of-concept RCT and main RCT were primarily informed by the target areas of the app's intervention and relapse criteria, but additional outcome measures were included based on qualitative work in phase 1. For example, participants often stated that the presence of a mental health smartphone app could be destigmatizing, so the potential for a stigma questionnaire was taken to an ERG meeting and approved by members as an outcome measure.

Participant recruitment can be a challenge in mental health research due to service and service user-related factors. For example, clinicians report barriers such as research saturation within services, negative prior experiences of research and paternalistic attitudes toward service user ability, safety or wellness (50). Service users have also reported barriers to participating in research such as transport difficulties, distrust of researchers, mental health-related stigma (51), lack of time and feeing too unwell or tired (52). Both the Actissist proof-of-concept RCT and powered RCT of efficacy recruited to target within the funding timeframes and the success of recruitment was helped by the strategies proposed by ERG members. For example, clinical members advised offering training incentives to mental healthcare staff working in NHS teams to help them feel that the time and effort spent on referring participants was worthwhile. Therefore, the principal investigator and Actissist team offered clinically relevant training to several NHS teams, which aided referrals. Likewise, clinical members also proposed that the Actissist team should ask mental healthcare staff to review therapy waiting lists to identify potential referrals. This strategy was extremely successful and resulted in 126 referrals (50 after consent and randomization). Members of the ERG with lived experience of psychosis advised on the recruitment materials, which aided the readability and attractiveness of the project for potential participants.

#### Striking a Balance Between Suggestions and Fundamental Features of the RCT Design

A challenge encountered during the involvement process was that some ideas regarding app design and functioning did not fit within the requirements of the RCT design. Tensions can sometimes arise in terms of what constitutes good quality research and what advisory groups feel would be acceptable (47). In these instances, it is important to negotiate what adjustments can be made to improve the app or trial procedures, whilst maintaining trial integrity. For example, the presence of pseudo-random alerts to engage with the app were highlighted as an improvement by participants in phase three exit interviews who requested that alert number and timings should be personalized based on user preference. However, the app we used in our active control condition (ClinTouch) delivers three pseudo-random alerts per day. Adapting the alert frequency on Actissist would result in the apps not being directly comparable; therefore, making such an adjustment was not compatible with a RCT design. However, based on the feedback received about the app alerts, future iterations of the app in non-trial settings would allow for the personalization or removal of the alert feature.

During the phase three exit interviews, some participants mentioned that it was difficult to provide in-depth feedback about the app because they were interviewed after their 22-week follow-up assessments (at least 10 weeks after using the app). Interviews were delayed due to the potential for an additional visit with a researcher impacting on final follow-up outcomes, potentially leading to inaccurate findings. As a result, participants suggested being able to provide in-the-moment feedback in the form of texts, app questions or emails to the Actissist team to provide more detailed responses to questions. Such procedural changes were discussed within the Actissist team and with ERG members; however, the inclusion of such options was viewed as having the potential to be detrimental to participants focusing on other areas of the app, thus potentially impacting study outcomes.

Automatic sharing of app data with clinicians was also viewed as a potential improvement to the Actissist app in the phase 1 and phase 3 interviews. ERG members also felt the app could be used to facilitate blended therapy in the future. It was decided that app data should not be shared with clinicians because some service users and clinicians felt uneasy about automatic data sharing and clinicians may respond to the data in varying ways, thus potentially affecting study outcomes. However, the Actissist team highlighted to participants during app set-ups that data would not be automatically shared with clinicians, but that they could show the app and graphical summaries to members of their care team themselves if they felt it would be helpful.

#### Providing a CBT-Informed App, Rather Than a Mental Health Toolkit

The core function of the Actissist app is to provide people with CBT-informed strategies to help them manage difficult experiences. Although other relevant aspects are included such as mindfulness, self-monitoring and psychoeducation, the uniqueness of Actissist is the CBT-informed question-answer exchange and strategies. Therefore, there was a balance between including everything that people wanted from Actissist, whilst maintaining the core function of the app. For example, many participants in the phase 3 exit interviews requested additional mindfulness and relaxation exercises, more interactive psychoeducation factsheets and further recovery videos. Some clinical ERG members also recommended intervention-related adaptions to the Actissist app, which were at odds with CBT principles. Such dilemmas were summarized by one service user in the phase 1 interviews who recognized that the app should not “get bogged down with too many features.” In line with the overall ethos of the app, suggestions about areas central to the CBT-informed content were prioritized over improvements to other areas. For example, ERG members suggested that the ability to set goals in the app was a priority area to include because they viewed goal-setting as a fundamental aspect of CBT. Additionally, we included links on the app to additional resources that could be used access suggested features. Examples included links to: (i) the Elefriends mental health forum for peer support (www.elefriends.org.uk); (ii) the NHS Get Active Your way website for advice on healthy living and exercise (www.nhs.uk/live-well/exercise/get-active-your-way); (iii) the Talk to Frank website for information about a range of substances (www.talktofrank.com); and (iv) the HealthTalk website for additional recovery and experience videos (www.healthtalk.org).

#### Hypothetical vs. Actual Acceptability

Service users and clinicians requested alerts in the phase 1 qualitative interviews and phase 2 beta-testing to help improve engagement with the Actissist app. The average number of alerts requested was between three and four. However, during the phase 3 interviews it was apparent that the number of alerts seemed too much to many participants and the number should be reduced or tailored. Such discrepancies in views highlight the issue that features initially viewed as positive and helpful when considered hypothetically may not translate to being acceptable in practice.

#### Limitations

Limited resources often associated with running an RCT can be a barrier to collaborative engagement with stakeholders. Meaningful collaboration can be costly and time-consuming, resulting in additional workloads for research staff who must also focus on participant recruitment and data collection (47). Due to limits in staffing levels, we were unable to reach out to as many service users as we had hoped to participate in ERG meetings. As a result, service user attendance at ERG meetings was sometimes variable and it was a challenge to maintain consistency with regards to member attendance. Member consistency has been identified as a challenge in other projects due to factors such as unavailability and perceived demands of the meetings (53). Similarly, a recent reflection on co-designing a research project with young people highlighted that although strong relationships were formed with young people who were involved in data collection and analysis were, it was harder to build such relationships with young people not involved in these areas of the project due to their role activity being intermittent (45). The delay between the Actissist proof-of-concept RCT finishing and main RCT starting made it difficult to maintain member consistency, resulting in different members taking part in the proof-of-concept RCT ERG and main RCT ERG meetings.

Resource pressures on digital health projects are not just limited to the research process and it is also the case that the software team has a fixed amount of staff resources available in order to deliver the DHI. It is inevitable that more ideas are generated by ERG members and qualitative participants than can be implemented within the time and funding constraints. In these instances, suggestions for changes had to be prioritized and compromises had to be made. A recurrent topic in our qualitative (phases 1 and 3) work was that participants wanted a range of additional areas covered in the CBT-informed “What's Bothering Me?” section. The “What's Bothering Me?” section works by asking users to select the area that they wish to work on (voices, paranoia, perceived criticism, socialization and cannabis), followed by four questions, the answers to which inform the subsequent therapeutic strategies provided. A number of different therapeutic strategies are available for each answer, which means that participants receive different suggested strategies each time they use the app, even if they give the same answers as their previous engagement. These processes are resource intensive to both the software engineering team programming the app and the clinical team designing the app content. Therefore, it was not possible to deliver the additional areas in a timely manner prior to trials commencing. As an alternative, we incorporated the additional topic areas proposed by building an “other” button in the “What's Bothering Me?” section, which directed users to additional psychoeducation factsheets that were created based on the requested topics.

Another common request from the qualitative interviews (phases 1 and 3), beta-testing (phases 2 and 4) and ERG members was the inclusion of additional multimedia resources. For example, recovery videos, multimedia options to present factsheets, a function to upload music into the app and a voice recording feature to allow users to speak directly into the daily diary were all viewed as helpful improvements. However, the space available on the app and technical complexity involved meant that it was only possible to implement one of these suggestions. It was decided that the development of additional recovery videos would be prioritized because a larger proportion of people requested this change. ERG members with lived experience of psychosis co-created these recovery videos with the Actissist team and the resulting videos were shown at an ERG meeting for member feedback.

### Recommendations

Based on our experiences of the involvement of stakeholders in the development and design of a mental health app for psychosis we make the following recommendations.

1. Increased funding for stakeholder involvement

We had funding allocated to stakeholder involvement in the ongoing development of the Actissist project and smartphone app. However, funding only allowed us to facilitate ERG meetings, pay individuals for beta-testing the app, and co-produce recovery videos. In future projects, we recommend a PPI expert is costed into grants either was a Co-investigator or as a paid role within the team. This would allow members to be frequently contacted with updates and activities, provided with further support in their role as a public contributor (for example, training in analysis methods) and facilitate continuity of members by ongoing communications. Increased stakeholder involvement using participatory research methods is also important to ensure greater and meaningful involvement of individuals with lived experience.

2. Virtual stakeholder engagement

In line with the ethos of the project, individuals were able to teleconference if they were unable to attend in person. However, this was not offered as standard and only available should a member request. Future projects should extend such opportunities to form virtual ERG meetings, which are unconstrained by location and may be less anxiety-provoking for individuals than attending in person. Conversely, the cost pressures associated with technology ownership, limited technical skills and anxieties about technology may prevent a diverse range of attendees. Potential ways to involve individuals who are unable to attend in-person should be identified during the early stages of project design, particularly in light of the COVID-19 pandemic, which has led to many projects needing to adapt to, and now embrace, PPI via remote methods.

3. Acknowledge what is possible with team skillsets

Feedback from participants, beta-testers and ERG members showed that version 1 of the Actissist app was straightforward to use, clear and user-friendly; however, it was also viewed as basic and functional in its design. UI design was undertaken by the software engineering team for version 1 of the app, but the team identified that a specialist UI designer would be needed to make the user-requested changes to improve the look-and-feel of the app (42). Such changes required flexibility in budgets and time and the acknowledgment that additional resources were needed to make suggestions possible. Therefore, individuals collaborating with stakeholders to develop DHIs must be mindful that flexibility in team dynamics is required to deliver what is important to stakeholders.

4. Identify what resources are already available

Many areas of improvement mentioned were related to the additional content on the app, rather than the CBT-informed “What's Bothering Me?” section. Despite some participants indicating that they did not engage with these areas of the app, those who did suggested that more of these additional resources should be included. When discussing the proposed changes within the Actissist team and ERG meetings and reflecting on how people said they already used technology in phase 1 interviews, we collaboratively agreed that there were sufficient resources already available to link to that meet these needs. When developing DHIs, researchers should discuss what is already available with stakeholders to identify whether this would be sufficient for their needs and think about ways to best incorporate links to such resources within the DHI.

5. Find compromises

We sometimes encountered situations where we were unable to provide what individuals asked for because of resource limitations. Feedback should be provided to stakeholders in a sensitive manner to reflect why such changes were not possible and compromises should be discussed to ensure that feedback is not tokenistic.

6. Involve multiple stakeholders

In Actissist we collaborated with a range of stakeholders, rather than solely individuals with lived experience of psychosis. We were concerned about the power imbalance that could be generated by a multidisciplinary group and were mindful of this potential tension when delivering the groups. From our own experience, the involvement of multiple stakeholders enhanced the development of the Actissist app as it generated a wider range of ideas and views than would have been possible through service users alone. These ideas were then assessed collaboratively by the clinical and software teams in order to create a development plan for the app. However, the current paper did not seek to explore individuals' experiences of participating in the ERG. Based on our experiences of running an ERG, we are not in the position to recommend whether this format works better than PPI groups and although we tried to mitigate any power imbalances associated with a multidisciplinary group, it is of note that some research staff at times felt intimated by the seniority of others attending the group. Therefore, we would encourage formal evaluation processes in future DHI research involving ERG meetings to understand the experiences of all individuals in attendance.

7. Develop bespoke subgroups

The ERG design was helpful for the overall development and design of the app and trial procedures. However, researchers should be mindful about specific topics or in-depth discussions that may be better suited to bespoke subgroups within the overall ERG network.

8. Adaptations need to be aligned with intervention theory and principles

In the digital health field, there is a tension between ensuring fidelity to trial procedures and the intervention being evaluated and evolving the technology to ensure it remains relevant by the end of the project period. Changes made to an intervention or trial procedure and adaptations made should be considered alongside fidelity to the trial (54). The Accelerated Creation-to-Sustainment (ACTS) model (55) is a framework for accelerating research and integrating design, evaluation, and sustainable implementation into a unified effort and warrants consideration when developing and evaluating digital health technologies.

## Conclusion

The involvement of stakeholders in the design, development and delivery of the Actissist app has been fundamental to our development approach. Through this collaborative process we have identified different perspectives and ideas that would have not been generated by the research team alone. We believe that the successes and challenges highlighted can be helpful for other researchers who want to achieve collaborative working when developing future digital health tools and systems.

## Data Availability Statement

The authors don't have permission to share the data. Requests to access the datasets should be directed to natalie.berry@manchester.ac.uk.

## Ethics Statement

The studies involving human participants were reviewed and approved by the National Research Ethics Committee West Midlands—South Birmingham (14/WM/0118) and National Research Ethics Committee West Scotland (17/WS/0221). The patients/participants provided their written informed consent to participate in this study.

## Author Contributions

NB wrote the first draft of the manuscript. SB, MM, DE, GH, SL, and RM wrote sections of the manuscript. All authors contributed to manuscript revision, read and approved the submitted version.

## Conflict of Interest

SB, JA and SL are directors of a not-for-profit community interest company spun out of the University of Manchester designed to make digital health apps commercially available in the UK National Health Service. The remaining authors declare that the research was conducted in the absence of any commercial or financial relationships that could be construed as a potential conflict of interest.
