# Supplementary material for: Developing a Theory-Informed Smartphone App for Early Psychosis: Learning Points From a Multidisciplinary Collaboration
Source: Front Psychiatry. 2020 Dec 10;11:602861. doi: 10.3389/fpsyt.2020.602861 (PMC7758439; doi:10.3389/fpsyt.2020.602861)
Supplement: Supplementary file 1 [file Data_Sheet_1.doc]

School of Psychological Sciences,

The University of Manchester

2^nd^ Floor, Zochonis Building

Brunswick Street

Manchester, M13 9PL

Tel: 0161 306 0400

Email: [sandra.bucci@manchester.ac.uk](mailto:sandra.bucci@manchester.ac.uk)

**Interview Guide – Phase 1a - Patients – Qualitative Interviews**

**Version 1 (updated)**

**Study title: Active Assistance for Psychological Therapy (ACTISSIST): Using mobile technology to deliver cognitive behaviour therapy in psychosis.**

Equipment- PIS-PATIENT interviews, Patient consent form, audio-recorder (x2), socialisation branching handout.

1. P.I.S- Any related questions?
2. Consent form- Explain audio recording and the limits of confidentiality. Care co-ordinator?
3. Demographic data (separate sheet)
4. General mobile health and engagement

Introduce self, welcome & thank participant for attending interview, ensure comfortable.

Re-confirm informed consent still valid.

Outline interview procedures, time duration, audio-recording, offer pauses, breaks, etc.

Explain limitations of confidentiality (ie research becomes aware of potential harm to self or others).

Explain purpose of the interview in relation to the ACTISSIST study:

*I am meeting with you today to ask you about your* ***views*** *of using* ***mobile phone technology*** *to provide* ***treatment for people*** *with psychosis. This is because we are going to design a mobile phone ‘app’ for people who have experienced psychosis. We would like your thoughts on* ***whether you think it’s a good idea, what would make people use the ‘app’*** *and so forth. We are going to ask you some specific questions about technology and then more specific information about he ‘app’ we are developing.*

*This interview will take about an hour. With your permission, the interview will be audio-recorded and then typed up so that we can make an accurate summary of what we discuss during this interview. Recordings will be kept locked away or in password protected computer files. Quotes from the interviews may be published but they will in no way be identifiable.*

***Just very briefly, what do you see the EIS for?***

***General technology in mental health questions***

I am going to ask some general questions about how you use technology, and then some specific questions about the ACTISSIST ‘app’. If there is any language I use that you are not clear about please let me know.

If own a SP, ***What do you use your SP for****?*

If no SP, ***If you had a SP what would you use it for****?*

*Do you use the internet to find out information, or to talk about, your mental health? (e.g. forums, Facebook, twitter, blogs).*

*If Yes, would you be able to tell me a bit about that? If No, what are your thoughts about using the internet to talk about your mental health problems?*

*What do you think about using an app to help you with any of the things you are seeing the EIS for? (e.g. helpful thing / unhelpful thing)*

*What do you think your relative/a close friend would think about you using a mobile phone app as a way of getting help for mental health problems (use client’s own words)?*

*Have you ever had a talking therapy? (e.g. talking to a psychologist, CBT)?*

*What are your thoughts about using an app to deliver a talking therapy (like CBT, if participant is familiar with this term)?*

*Not everyone gets access to the help that they need. Is a mobile phone app a way of overcoming issues related to access for help with mental health problems?*

*(In what way? Can you tell me more about that?)*

*Can you see an app helping you with the reason why you are seeing the EIS? Would you want to use an app for this? Would you want to do this instead of talking to someone?*

*Would you consider using your mobile phone to inform people that you might be becoming unwell?*

**Introduce and summarise Actissist**

*How would you feel about receiving a talking therapy over your/a Smartphone?*

*How would you feel about answering questions four times a day, 6 days a week for about 3 months? (Is this OK/too frequent/not frequent enough?)*

*What would get in the way of you being able to answer questions on the app?*

Treatment Domains

*How would you feel about answering questions each day about your mental health (ensure that information is gathered for each treatment target domain – read these out separately rather than in a list):*

*- activity levels (make it clear it’s not about exercise) - pause*

*- whether you’re feeling criticised by a person you are close to/who is important to you, drug us (if relevant) - pause*

*- symptoms (voices, worries) - pause*

*- medication?*

*Are these the right areas the app should be targeting? Are there any important areas that we have missed?*

Branching questions

*How would you feel about responding to a series of prompts/questions, which would then make recommendations about which treatment you receive in the app? Would you want the app to recommend information to you, or would you prefer to choose this yourself?*

Show socialisation branching handout:

*How do these questions look to you? Do they make sense? Would you want to answer them? How could we improve the way we ask questions?*

*About how long would you want to spend replying to messages (per beep)?, And in total (per day)?*

Repository

*We would like to include a resource section in the app. It will include things like relaxation exercises, mindfulness exercises, a thought diary, video of someone’s recovery journey, links to music and video material.*

*Does this sound like something you would use?*

*Is there any other information that you would like to see in the app?*

*Is there anything important that we have missed?*

*Do you have any concerns about the examples of resources in the app?*

Summary Data

*We were thinking that each week we would send people a summary of information they entered into the app. What are your thoughts about this? Would it be helpful/not helpful?*

**ACTI- TheVirtual Therapist**

**Good idea/ bad idea? What should the look like? Human/non-human?**

General Actissist-related questions

*Would you try using this app if it was part of the care offered by the EIS?*

*What could we do to make sure that people got involved in using the app?*

*Are there any problems you can foresee with using an app like the one I described?*

*Any ideas about how we could overcome these problems?*

*What problems might there be in using this app?*

*If you have someone who helps you look after your mental health (e.g. partner, friend, parent, sibling), would you want them to be involved in using this app? If so, how?*

*What do you think the key features of the app should be/what aspects of mental health should the app target?*

**Privacy concerns**

*Do you have any concerns about using a mobile phone in general?*

*Do you have any concerns about using a mobile phone for your mental health problem (use participant’s own words)?*

*Do you think using a mobile phone app could have any negative effects? Does it worry you?*

*If you do have any concerns, how could we reassure you that the app was safe?*

*Would you worry about other people having access to the information in your app?*

**Closing prompts**

*Is there anything else you would like to tell me that we’ve not talked about but might be important for me to know about how to improve the ‘app’?*

*How have you found being involved in this interview today? (offer reassurance if needed, allay concerns)*

*How might we improve the experience for other participants?*

**End interview, thank participant, explain what will happen to the information discussed, offer to provide summary of study findings when available. Invite to be a member of the ERG.**

**Can we contact you again when we have developed the app and are ready to test it out / run the RCT?**
