# Supplementary material for: Developing a Theory-Informed Smartphone App for Early Psychosis: Learning Points From a Multidisciplinary Collaboration
Source: Front Psychiatry. 2020 Dec 10;11:602861. doi: 10.3389/fpsyt.2020.602861 (PMC7758439; doi:10.3389/fpsyt.2020.602861)
Supplement: Supplementary file 2 [file Data_Sheet_2.doc]

School of Psychological Sciences,

The University of Manchester

2^nd^ Floor, Zochonis Building

Brunswick Street

Manchester, M13 9PL

Tel: 0161 306 0400

Email: [sandra.bucci@manchester.ac.uk](mailto:sandra.bucci@manchester.ac.uk)

**Interview Guide – Phase 1a - Staff Focus groups**

**Version 1**

**Study title: Active Assistance for Psychological Therapy (ACTISSIST): Using mobile technology to deliver cognitive behaviour therapy in psychosis.**

*What follows is a guide: The order and exact content of the questions will be determined by the participant and will be influenced by the ongoing analysis so the order of the questions may vary as the interview develops.*

*Probe and ask for examples as the time permits.*

Introduce self, welcome & thank participant for attending interview, ensure comfortable, offer drink etc.

Re-confirm informed consent still valid.

Outline interview procedures, time duration, audio-recording, offer pauses, breaks, etc.

Explain limitations of confidentiality (ie research becomes aware of potential harm to self or others).

Explain purpose of the interview in relation to the ACTISSIST study:

**Introduction**

*We are meeting with you today to ask about your views of using mobile phone technology to deliver psychological interventions in early psychosis because we are developing a CBT-informed mobile phone app for people with first episode psychosis. This focus group will take 1-1.5 hours. With your permission, the group discussion will be audio-recorded and then transcribed so that we can make an accurate summary of what we discuss during this group. The transcription will be given an ID number and will be anonymised so that no identifying information appears.*

**Work and workload**

*Ask staff members to tell you about their role. How long they have been doing it and what their main responsibilities are, etc.*

- Can you tell me about your role, how long you have been doing it and what are your main responsibilities?
- How many service users do you support and how do you support them?
- How often do you have contact with them and visit them?
- Can you tell me about any barriers to performing your job effectively?
- What are our views about current provision of mental health services for people with serious mental illness?
- Do you see technology playing a role in the provision of mental health services? What about in delivering psychological therapy, such as CBT?
- How do you think using mobile technology might impact on your workload?

**Supporting self-management**

- Do you think there are any existing barriers or problems in detecting signs of deterioration in your service users?
- Are there any barriers in working effectively with your service users?
- Do you think your service users have an active approach to self-management?
- What are your views on self-management?
- What do you think are the main barriers to good self-management for service users?

**General views about using technology to deliver CBT in early psychosis**

- What were your immediate impressions of the idea of ACTISSIST? What were your views about using an app to deliver a talking therapy?
- What resources, other than mental health services, do your service users use to help them manage their mental health (e.g. friends, family, internet, mobile phone, etc.)?
- Do your service users use technology to monitor their symptoms, gather information about mental illness, etc.?
- What are your views on using technology to deliver psychological therapy (CBT) for early psychosis?
- 1/10 people who should have access to CBT get access to this therapy. Do you think technology could overcome these access issues for people with psychosis?
- Do you think your service user’s carers/relatives would support their family member in using a mobile app delivering a CBT intervention for psychosis?
- Do you see a mobile app successfully delivering CBT helping people with psychosis? How so?
- Do you see a mobile app delivering CBT adversely effecting your service users? How so?
- How do you think using an app like Actissist could improve self-management for people with mental health problems?

**Introduce and summarise Actissist (refer to information sheet)**

- Do you think delivering CBT in this way could be useful in early psychosis management?
- Do you think people with psychosis would be receptive to receiving CBT delivered using their mobile phone?
- Do you think your service users would respond to an app that asked questions about perceived criticism, drug use, symptoms, socialisation and medication four times a day, 6 days a week for 12 weeks?
- In your view, is this time frame too much/not enough?
- Do you think an app like this would be useful in supporting service users with psychosis?
- What kinds of problems could you foresee in using this app?
- How do you think this app might impact on your workload?
- How might this app improve the quality and effectiveness of service user’s care?
- How do you think your service users would feel about using this app?
- What do you think would encourage service users to use this app?
- What kind of incentives do you think might encourage service users to keep using the app for the duration of the trial period?
- What are the key features the app should have?

**Privacy concerns**

- Based on your knowledge and experience, do you have any concerns or do you foresee any problems regarding the use of ACTISSIST?
- Do you have any concerns regarding privacy, security of data or other safety issues?
- Do you think it is acceptable to deliver CBT to people using this kind of system and do you think people would mind receiving psychological treatment in this way?
- Do you think using a mobile app would have any negative effects on your client’s mental health (e.g. increase paranoia, make symptoms worse)?
- What could we do to reassure you/your clients about the safety of their information?

Is there anything else you would like to tell me that we’ve not talked about but might be important for me to know about how to improve the ‘app’?

Finally may I ask how you have found being involved in this interview?

**End interview, thank participants, explain what will happen to the information discussed, offer to provide summary of study findings when available.**
