# Supplementary material for: Developing a Theory-Informed Smartphone App for Early Psychosis: Learning Points From a Multidisciplinary Collaboration
Source: Front Psychiatry. 2020 Dec 10;11:602861. doi: 10.3389/fpsyt.2020.602861 (PMC7758439; doi:10.3389/fpsyt.2020.602861)
Supplement: Supplementary file 3 [file Data_Sheet_3.doc]

School of Psychological Sciences,

The University of Manchester

2^nd^ Floor, Zochonis Building

Brunswick Street

Manchester, M13 9PL

Tel: 0161 306 0400

Email: [sandra.bucci@manchester.ac.uk](mailto:sandra.bucci@manchester.ac.uk)

**Interview Guide – Phase 3 - Patients – Qualitative Interviews (ACTISSIST arm)**

**Version 1**

**Study title: Active Assistance for Psychological Therapy (ACTISSIST): Using mobile technology to deliver cognitive behaviour therapy in psychosis.**

*What follows is a guide: The order and exact content of the questions will be determined by the participant and will be influenced by the ongoing analysis so the order of the questions may vary as the interview develops.*

*Probe and ask for examples as the time permits.*

Introduce self, welcome & thank participant for attending interview, ensure comfortable, offer drink etc.

Re-confirm informed consent still valid.

Outline interview procedures, time duration, audio-recording, offer pauses, breaks, etc.

Explain limitations of confidentiality (i.e. research becomes aware of potential harm to self or others).

Explain purpose of the interview in relation to the ACTISSIST study:

- Explore experience of using mobile phones / apps in the past

Using the mobile phone app (ACTISSIST)

- How did you find using the ACTISSIST app to manage your mental health?
- Did you have any difficulties using the phone or the app in the beginning?
- Have your views about the app changed since the previous interview? What has changed your mind?
- Do you think the app works for the purpose intended?
- Have you made any changes to how you respond to it during the time period?
- Do you think this is a good way to manage symptoms for people with early psychosis?
- Do you feel you had enough information to help you to use the app?

Completing the questions and using the repository of personally meaningful multi-media content (RPMM)

- Which questions/modules were most helpful? Is there anything you would have liked to have seen more of (e.g. more information on psychosis, etc.)?
- Were any questions more difficult than others?
- If there were questions you did not answer, can you give any reasons why?
- What did you think about the number of questions asked and how often they came?
- Is there anything we could have done to make it easier for you to answer the questions?
- How could we make each question as meaningful for you as possible?
- What could we do when you respond to questions to make responding more worthwhile?
- How motivated did you feel completing the prompts? Did you find aspect of the app annoying/unhelpful, etc.?
- Did you use the app only when it beeped, or did you use it at other times during the day?
- Did you do any of the relaxation exercises, access video clips, etc? Can you tell me more about that?
- Did you personalise your app with photographs and colour?
- Did you refer to summary graphs? Were the graphs helpful?

Fitting in with everyday life

- How well did using the phone/app fit into your everyday life? Has it changed anything that you usually do?
- How much time did you spend using it?
- Where did you keep it?
- Were you aware of it, even when you are not using it?
- Did any of the messages wake you up?
- Were there any times when the beeps interrupted what you were doing? If so, what was it that you were doing?
- Did you ever forget to take the handset out with you?
- Have you shown it to anyone else or discussed it with anyone else? What were their views?
- If you were prompted to complete the questions whilst with other people, did you tell them about it?
- Did you feel like using the phone had any impact on your relationships with other people?
- Did you feel that using the phone ever felt part of your normal routine?
- How long do you think you could or would use the app for? Do you think 12 weeks is too long / not long enough?

Impact on managing mental health

- Has using the app made any changes to the way you manage your mental health?
- Do you think you are aware of your mood and symptoms more now than before using the phone?
- Has using the phone changed the way in which you think about your difficulties?
- Has it changed the level of confidence you have to manage your mental health problem?
- Explore views of the most important outcomes of therapy (app).
- Identify views of any benefits or difficulties as a result of participating in ACTISSIST.

Benefits and problems

- Were there any benefits to taking part in the study?
- Were there any negative consequences for taking part in the study?
- Is there anything you would change about the procedure?

What could be improved?

- Are there any ways in which the app could be improved?
- Privacy and Safety - did you feel that the app was safe? Did you have any privacy concerns while using the app?
- What could we do to assure you that your information is safe?

Is there anything else you would like to tell me that we’ve not talked about but might be important for me to know about how to improve the ‘app’?

Finally may I ask how you have found being involved in this interview?

*Prompts*: How might we improve the experience for other participants?

**End interview, thank participant, explain what will happen to the information discussed, offer to provide summary of study findings when available.**
