# Supplementary material for: Developing a Theory-Informed Smartphone App for Early Psychosis: Learning Points From a Multidisciplinary Collaboration
Source: Front Psychiatry. 2020 Dec 10;11:602861. doi: 10.3389/fpsyt.2020.602861 (PMC7758439; doi:10.3389/fpsyt.2020.602861)
Supplement: Supplementary file 4 [file Data_Sheet_4.docx]

**
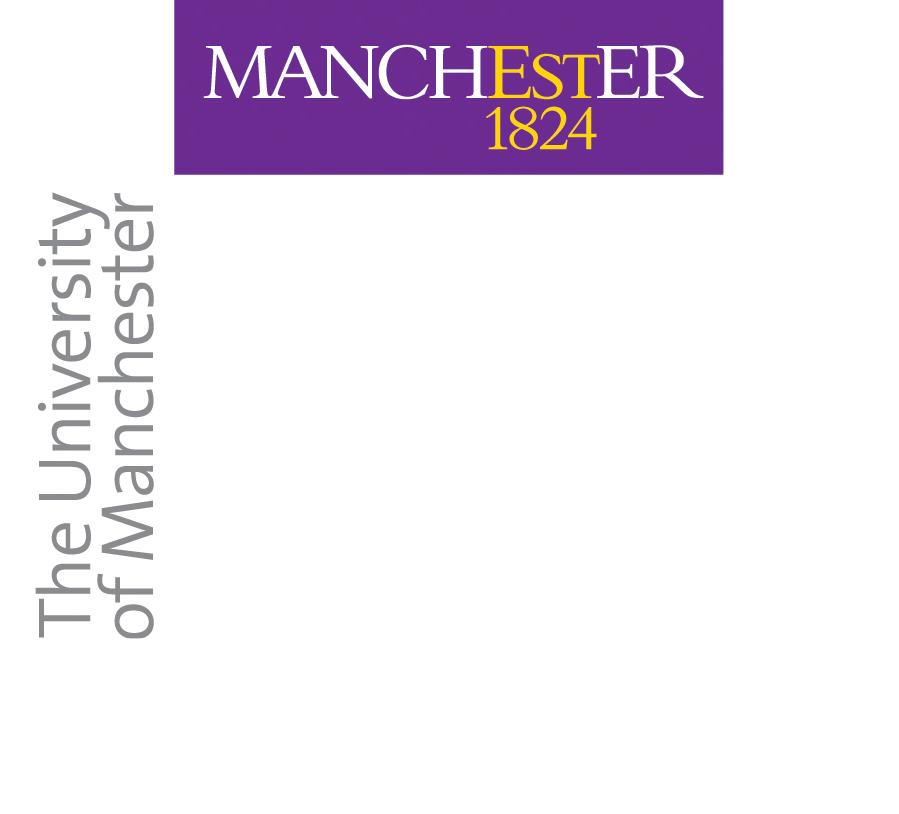

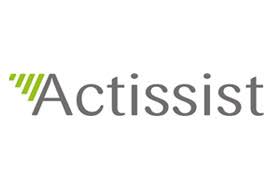
**

**Actissist**

**Summary of feedback from Actissist qualitative interviews, focus groups and ERG meetings**

**Suggestions for changes and improvements**

**Key:**

Likely to be able to rectify as soon as possible

Might be able to rectify as soon as possible

Long-term/eventual changes

Continually addressed as project progresses

Concern highlighted likely to be due to personal preference and not reported by other participants - take to expert reference group

| **Area identified and example quotes** | **Potential solutions** | **Completion stage** |
| --- | --- | --- |
| **Functionality** | |  |
|  |  |  |
| **Question phrasing** | |  |
|  |  |  |
| **Presentation of information** | |  |
|  |  |  |
| **Content** | |  |
|  |  |  |
| **Daily Diary** | |  |
|  |  |  |
| **Alerts** |  |  |
|  |  |  |
| **Appearance** |  |  |
|  |  |  |
| **Procedural improvements and difficulties in finding specific information** | |  |
|  |  |  |
| **Involvement of health care professionals** |  |  |
|  |  |  |
